# Effects of Water Extract of *Cynanchum paniculatum* (Bge.) Kitag. on Different Breast Cancer Cell Lines

**DOI:** 10.1155/2021/6665949

**Published:** 2021-05-19

**Authors:** Shu-Yu Yang, Jen Ying Li, Guan-Jhong Huang, Badrinathan Sridharan, Jen-Shu Wang, Kai-Ming Chang, Meng-Jen Lee

**Affiliations:** ^1^Department of Chinese Medicine, Taichung Tzu Chi Hospital, Buddhist Tzu Chi Medical Foundation, Taichung, Taiwan; ^2^Department of Chinese Pharmaceutical Sciences and Chinese Medicine Resources, China Medical University, Taichung, Taiwan; ^3^Department of Applied Chemistry, Chaoyang University of Technology, 168 Jifeng East Road, Taichung, Taiwan; ^4^Department of Research, Koo Foundation Sun Yat-Sen Cancer Center, 125 LihDer Road, Pei-Tou District, Taipei, Taiwan

## Abstract

*Cynanchum paniculatum* (Bge.) Kitag. (CP) is an important medicinal herb used in Chinese herbal medicine, with a variety of biological activities including anticancer property. In this study, we explored the water extract of CP, for its anticancer effects against breast cancer cells with different mutation types. Cells were grouped as untreated (Control); CP direct treatment (dir-CP); Conditioned medium from CP treated (sup-CP), and untreated cells (sup-Control). Effects of dir-CP and sup-CP were compared to corresponding untreated cells on cytotoxicity, cell migration, and protein expression (cleaved caspase-3, caspase-9, and MMP-2 and 9). CP treatment showed time-dependent decrease in cell number of MDA-MB-231 and SK-Br-3 (both ER(−) PR(−)), while the decrease in cell number was not as significant in MCF-7 and ZR-75-1 cells (both ER(+) PR(+)). sup-CP treatment inhibited the cell migration of MDA-MB-231 and MCF-7 (Her2(−)) in a 24 h scratch assay. Our data suggested that ER(−) PR(−) cells are more sensitive to the CP in terms of direct cytotoxicity, which is not regulated by caspase-3. CP inhibited the migration of the two Her2(−) cells, and this correlated with MMP-2 regulation. The migration of ER(−) PR(−) cells was more sensitive to conditioned medium with CP treatment than to direct CP, and this is not regulated by MMP-2. Our data suggested that CP has anticancer potential on various breast cancer cells through different mechanisms and is specifically effective in inhibiting the migration of the triple negative MDA-MB-231. Our data provide insight into the mechanism of CP against breast cancer progression and would benefit the medical practitioners in better management with CP usage.

## 1. Introduction

*Cynanchum paniculatum* (Bge.) Kitag. (CP) (also known as dog strangling vine, radix cynanchi paniculata, Shu Changching in Chinese, SCC) belongs to the genus of Cynanchum, a genus of about 300 species including some swallow-worts, and the family Apocynaceae. They have slender and rigid stems which can grow about 1 meter and the roots are densely fibrous. The root and stem were traditionally used in Chinese medicine for symptoms including pain, arthritis, itching, swelling, and blood smoothing [1]. The 992 C.E. classical Chinese medicine book “Taiping Shenghui Fang” (the Taiping Holy Prescriptions for Universal Relief) described the usage of Shu Changching (i.e., *Cynanchum paniculatum* (Bge.) Kitag.) for curing heart ache and malicious ulcer, which indicated breast cancer. In modern Chinese medicine practice, it was used in conjunction with other medicinal plants for cancer [2, 3]. However, the mechanism of the action exerted in the treatment of cancer or other ailments by this plant extract is still under exploration [4, 5].

Breast cancer comprises the second largest population of cancer patients worldwide and is one of the important causes of death among women. The standard treatments for breast cancer have evolved from surgery, chemo/radiotherapy to antibody-based targeted therapy, due to the progress in cancer genomics. For example, among the early occurring population, patients with Her (+) overexpressing cells had benefited from the target drug Herceptin [6]. Her2(+) breast cancer cells are highly metastatic in nature and Herceptin is a recombinant monoclonal antibody that is targeted towards growth factor receptors (HER) in Her2(+) metastatic breast cancer cells. On the other hand, patients with breast cancer that are triple negative (ER(−) PR(−) Her-2(−)) constitute about 15–20% of the breast cancer population lack Her2 gene and the specific disease metastasis with high recurrence rate challenges the medical experts in the management of the disease drug cannot be used for treatment [7]. These patients are generally younger and show a faster progression of the tumor which makes it very difficult to treat them [8]. For these patients, although other types of molecular target drug such as antiangiogenesis or immune therapy drug targeting the T cell checkpoint provide satisfactory results, the need for new chemical compounds that could act on this specific type of cancer still remains unattended [8, 9]. We intended to test whether CP extract could inhibit the proliferation of MDA-MB-231 cells (along with other breast cancer cells with different genetic variants) and also deduce the mechanism of cytotoxicity.

Several compounds were isolated from CP [2, 3, 10, 11] and two of them include paeonol [12, 13] and vanillic acid, which were among the components of the phenolic extract of food/medicine that tested positive for cancer cytotoxicity [14–16]. In this paper, we explore the action of water extract of CP on 4 different types of genomic compositions of breast cancer cells (for details, see Materials and Methods). The study intended mainly to explore and compare the mechanisms by which CP exhibit its antiproliferative and antimetastatic potential on different breast cancer cells that vary by their genomic profile. Chemoresistance of cancer to molecular targeted therapy due to repeated administration is an important hurdle to overcome for oncologists and researchers by the exploration of new drugs. In this paper, we study the direct effect of CP and its conditioned medium on four different mutant types of breast cancer cells, wishing to pinpoint the molecular mechanism that could be affected.

## 2. Materials and Methods

### 2.1. Breast Cancer Cells and Their Genomic Composition

The human breast cancer cell lines MDA-MB-231, MCF-7, and ZR-75-1 were obtained from the BCRC (Bioresource Collection and Research Center, Hsinchu, Taiwan), whereas SK-Br-3 were obtained from the American Type Culture Collection (Manassas, VA). Their phenotypes for estrogen receptor (ER), progesterone receptor (PR), and human epidermal growth factor receptor 2 (Her2) are listed in Table 1.

### 2.2. Collection of CP Plant Material and Water Extraction

A voucher specimen of *Cynanchum paniculatum* was deposited in the Department of Applied Chemistry, Chaoyang University of Technology, and given a code “CY0001”, as advised by the Herbarium of National Research Institute of Chinese Medicine, Ministry of Health and Welfare (MOHW), Taiwan. Dry Xu Changqing (100 g) was extracted with 300 mL water, at 100°C for 1 hr, at 1 atm. They were extracted twice and pooled and then dried with a freeze dryer. The weight of the dry paste was 20% of the starting material.

### 2.3. Culture of Cells

The MDA-MB-231 cells were cultured in Leibovitz's L-15 Medium (Gibco Life Technologies, NY, Grand Island), supplemented with 10% fetal bovine serum, in free gas exchange with atmospheric air. The MCF-7 cells were cultured in Eagle's Minimum Essential Medium (Gibco Life Technologies, NY, Grand Island) supplemented with 0.1 mM nonessential amino acids, 1.0 mM sodium pyruvate, and 10% fetal bovine serum. The SK-Br-3 cells were cultured in McCoy's 5a Medium Modified (Sigma-Aldrich, St Louis, MO), supplemented with 10% fetal bovine serum. The ZR-75-1 cells were cultured in RPMI-1640 Medium (Gibco Life Technologies, NY, Grand Island) supplemented with 4.5 g/L glucose, 10 mM HEPES, 1.0 mM sodium pyruvate, and 10% fetal bovine serum. The 184A1 cells were cultured in serum-free mammary epithelial basal medium (MEBM) supplemented with Bovine Pituitary Extract (BPE) 52 ug/ml, hEGF 10 ng/ml, Insulin 5 ug/ml, hydrocortisone 0.5 ug/ml, transferrin 10 ug/ml, using SingleQuot reagent packs from Lonza. Except for MDA-MB-231, the cells were cultured in a water-saturated atmosphere of 5% CO_2_ and 95% atmospheric air at 37°C.

### 2.4. Crude Extract Treatment

The cells were plated at the density of 10^4^ cells in 96 wells for cytotoxicity or plated at the density of 7 × 10^4^ cells in 24 wells using culture inserts (Idibi, Martinsried, Germany) specially made for the scratch assay. The cells were plated in the respective medium with 10% serum until almost confluent. They were washed with PBS and changed to 5% exosome-free FBS in their respective medium for 24 hours. Then the cells were supplemented with medium without FBS and the various concentrations of CP extract for 24 hours before carrying out the cytotoxicity test using CCK8 kit or removing the culture insert. This treatment was referred to as direct-CP and different from those treated with conditioned medium (or sup-CP) which will be discussed in detail below.

### 2.5. Collection of Conditioned Medium and Treatment to the Naïve Cells

**Figure 1:**
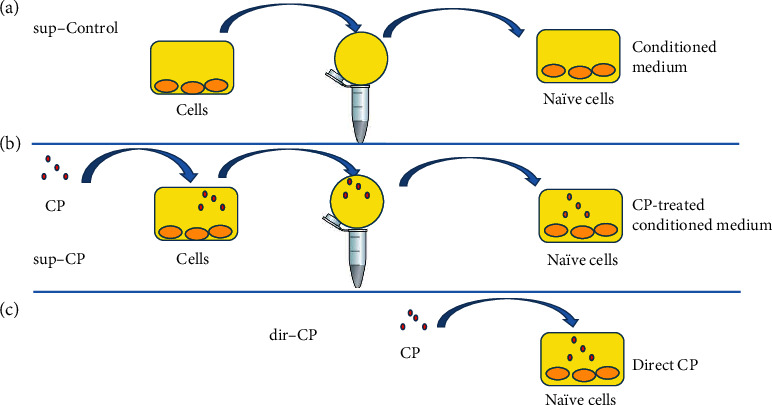
Schematic diagram for preparation of CP conditioned medium and treatment of CP extract and CP conditioned medium to the cells. (a) Conditioned medium without CP extract treatment was collected as a conditioned medium control (sup-Control); (b) Some cells were treated with the CP extract and the 24-hour conditioned medium was collected and the conditioned medium was used to treat naïve cells of the same cell types (sup-CP); (c) for the crude extract treatment (dir-CP), the naïve cells were treated directly with CP. The naïve cells were plated in the respective medium with 10% serum until almost confluent, washed with PBS, and changed to 5% exosome free FBS for 24 hours, and changed to medium without FBS and CP for 24 hours.

To test whether the certain effect was regulated by the secreted factors in the conditioned medium by an autocrine mechanism, some cells were treated with the CP extract as above described, and the 24-hour conditioned medium was collected, to treat naïve cells of the same cell types. The naïve cells were plated and treated like those for the direct CP extract treatment, but the CP extract was replaced by the conditioned medium (see Figure 1). A conditioned medium without CP extract treatment was collected as a conditioned medium control (or sup-Control).

### 2.6. Proliferation/Cytotoxicity Assay

The cells were plated at the density of 10^4^ cells in 96 wells until confluent and changed to CP extract containing serum-free medium for 24 hours before testing with a WST based proliferation/cytotoxicity assay using the CCK8 kit (cat no B34304, Bimake, Houston, USA). Four groups were tested, which include (1) the one treated directly with the CP extract (dir-CP), (2) cells treated with conditioned medium collected from the 24 hour CP extract treated cells (CP-sup), (3) cells treated with conditioned medium collected from the 24-hour incubation without CP extract (sup-Control), and (4) cells without any treatment (control).

### 2.7. Scratch Assay and Analyses of Cell Migration

The cells were plated at the density of 7 × 10^4^ cells in 24 wells using culture inserts (Ibidi, Martinsried, Germany) until confluent in respective medium supplemented with 10% FBS and changed to CP extract added in 5% exosome-free FBS medium while the insert was removed. The gap left after removing the insert, which resembled the scratch, was photographed at 24 hours, 48 hours, 72 hours, and 5 days after the removal of insert treatment. The four treatment groups were the same as those for the cytotoxicity assay. The cell migration was calculated as follows.

A photograph was captured by the CCD camera and the border of the scratch was determined from the time zero photograph. Lines were drawn by connecting the location of the cells and the right or left border (whichever is nearer). The distance of migration was estimated by measuring the length of this line in the ImageJ software (NIH freeware). The average distance of all cells in the fields was calculated and compared among groups.

### 2.8. Western Blots

The cells were trypsinized and the cell pellet was collected. The cells were then lysed in RIPA buffer (cat no AI0011, Able-Bio) containing a protease inhibitor mix, and the supernatant was collected. The culture medium was collected and passed through 3 kDa flow-through column (cat no UFC900396, Millipore, Bedford, MA) to desalt and concentrate the protein. They were mixed with loading buffer to make final concentration of 2.5 *µ*g/*µ*l∼5 *µ*g/*µ*l. Before immunoblotting, the cell lysate was boiled in a loading buffer with *β*-mercaptoethanol for 5 min. The cell lysates were separated on a 10%–12% SDS-PAGE and transferred to a polyvinyl difluoride (PVDF) membrane. The PVDF membranes were incubated in 0.1% milk in PBST (phosphate-buffered saline with 0.1% Tween-20) for 1 h to rid them of background staining, followed by overnight incubation in the primary antibodies at 4°C. Primary antibodies used were anti-MMP-9 (cat. nos. 3852; Cell Signaling Technology, Inc.), anti-MMP-2 (cat. no 13132; Cell Signaling Technology, Inc.), anticaspase-9 (cat. no 9502; Cell Signaling Technology, Inc.), anticleaved caspase-3 (cat. no 9661; Cell Signaling Technology, Inc.), and antiactin (cat. no. 4970; Cell Signaling Technology, Inc.). The secondary antibody used was anti-rabbit IgG conjugated with horseradish peroxidase (HRP) (Amersham) for all the proteins. After washing, blotted proteins were visualized using a Western blotting detection system (ECL Plus, Amersham, UK) and proteins bands were detected using Western Lightning™ Chemiluminescence Reagent Plus (Amersham Biosciences, Arlington Heights, IL, USA). Actin was stained and used as a normalization standard. After this normalization, each lane within the same blot was normalized against the control and the statistics were performed using this ratio.

### 2.9. Statistical Analyses

**Figure 2:**
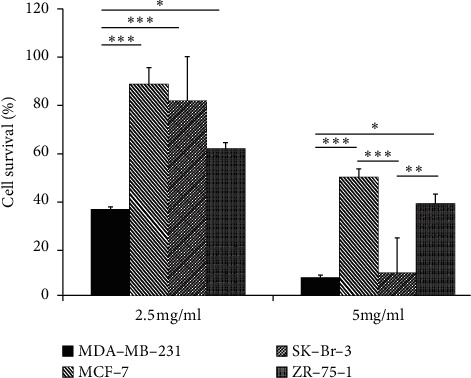
Cytotoxicity of CP extract on different cell types of breast cancer. The results were expressed as % survival of cells and were statistically analyzed by post hoc pairwise comparisons following the Kruskal–Wallace test: Dunn's method. The experiments were performed independently in replicated assays (*N* = 4) and comparisons were made between the cells. ^*∗∗∗*^−*p* < 0.001; ^*∗*^−*p* < 0.05.

**Figure 3:**
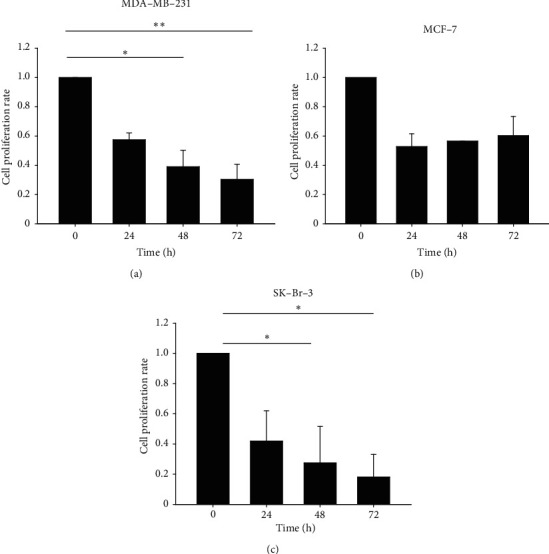
Time dependent cytotoxicity of CP extract on different cell types of breast cancer. The LC_50_ of CP extract on different cell types were determined and used for time dependent assay. The concentrations of CP treated are MDA-MB-231: 2.7 mg/ml, MCF-7: 3.1 mg/ml, and SK-Br-3: 3.05 mg/ml. Cytotoxicity was tested using the CCK8 kit after treatment of CP for 24, 48, and 72 hours. The results were statistically analyzed by nonparametric Kruskal–Wallis test for ANOVA and Dunn's post hoc pairwise comparison method. ^*∗*^^*∗*^−*p* < 0.01; ^*∗*^−*p* < 0.05.

**Figure 4:**
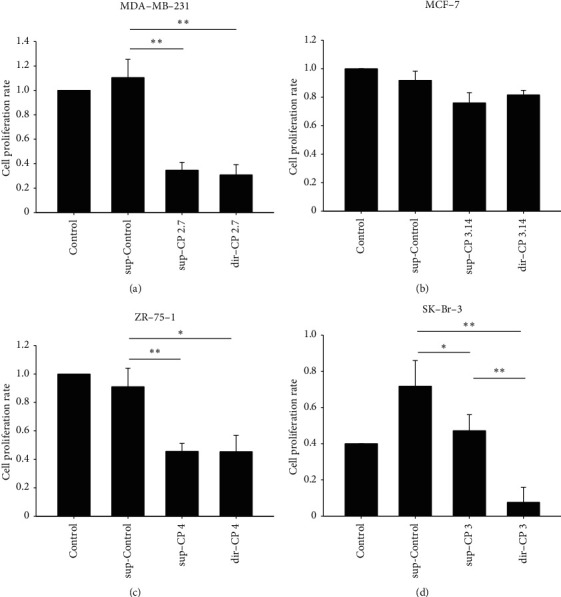
Cytotoxicity of sup-Control and sup-CP on naïve breast cancer cells. sup-Control and sup-CP preparation were prepared as explained in Figure 1. The results were statistically analyzed by post hoc pairwise comparisons following Kruskal-Wallis test. The experiments were performed independently in replicated assays (*N* = 4). ^*∗*^^*∗*^^*∗*^−*p* < 0.001, ^*∗*^^*∗*^−*p* < 0.01, and ^*∗*^−*p* < 0.05.

**Figure 5:**
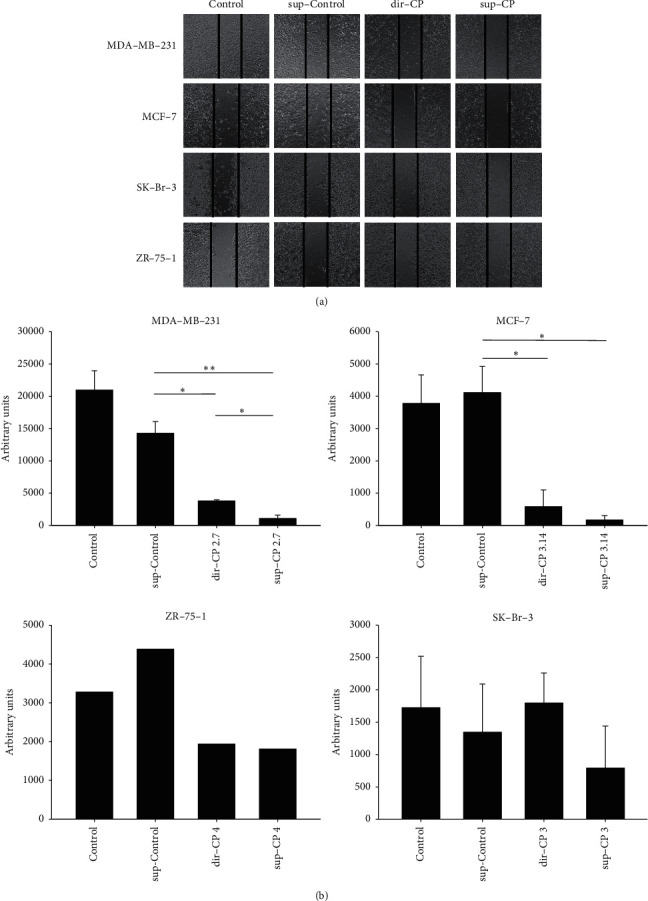
Scratch assay to study the migration of different types of cells treated with sup-Control; sup-CP & CP (dir-CP). The results were statistically analyzed by post hoc pairwise comparisons following Kruskal–Wallis test: Dunn's method and comparison was made as untreated vs. treated samples. The experiments were performed independently in replicated assays (*N* = 4). ^*∗*^^*∗*^−*p* < 0.01; ^*∗*^−*p* < 0.05.

**Figure 6:**
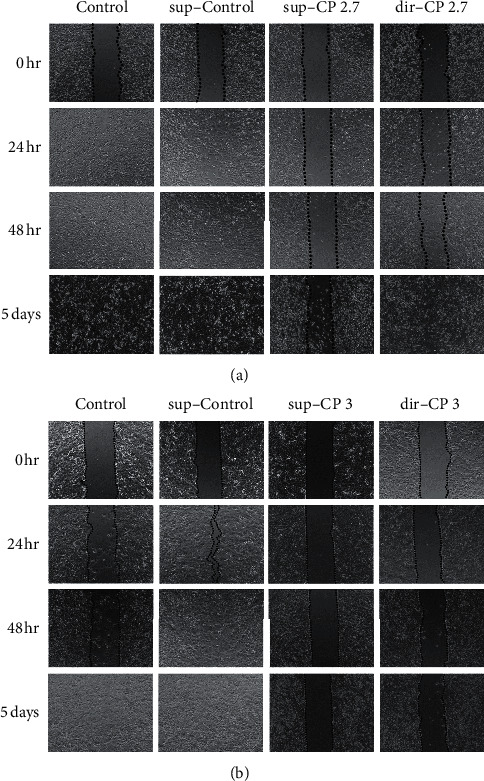
Scratch assay to study the time dependent migration of different types of cells treated with sup-Control; sup-CP, and CP (dir-CP). (a) is for MDA-MB-231 cells treated with 2.7 mg/ml of CP, and (b) is for MCF-7 cells treated with 3.14 mg/ml CP. The photos were taken at the beginning of various CP treatments (0, 24, and 48 hours and 5 days after treatment).

**Figure 7:**
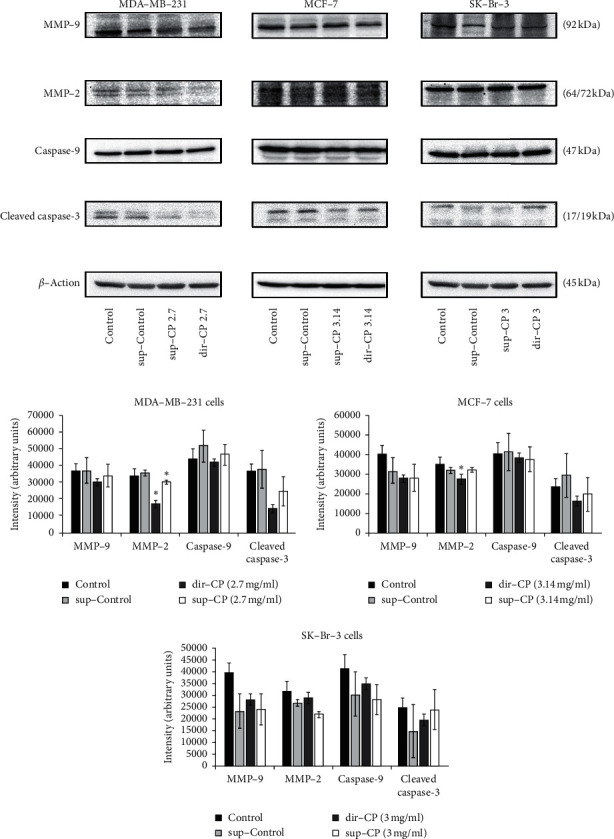
Immunoblotting studies of MMP-9, MMP-2, caspase-9, and cleaved caspase-3. Protein bands of different cell types of breast cancer treated with sup-Control, sup-CP, and dir-CP. The intensity of the bands was measured using ImageJ software. The results were statistically analyzed using post hoc pairwise comparisons for equal variance, i.e., Tukey's method, and the comparison was made as untreated vs. treated samples. The experiments were performed independently in replicated assays (*N* = 4). ^*∗*^−*p* < 0.05.

All the results obtained were analyzed with appropriate statistical tools. All the datasets were tested for normality using the Shapiro–Wilk test. One-way ANOVA was performed using Kruskal–Wallis test followed by post hoc pairwise comparison (Dunn's test) for Figures 2–4. In case of Figure 5, one-way ANOVA using Welch F-test was used followed by post hoc pairwise comparison (Tukey's method).  Figure 6 was not analysed by statistical analysis and Figure 7 was also analysed by one-way ANOVA using Welch F-test which was used followed by post hoc pairwise comparison (Tukey's method).

## 3. Results

### 3.1. LC_50_ of the Crude Extract to the 4 Cancer Cell Lines

We tested the LC_50_ for each cell line with a crude concentration range from 10 mg/ml to 0.01 mg/ml over 10 dilutions and plotted the survival rates over the concentrations. Linear regression was done using the 3–5 data points surrounding the 50 percent survival rates, and the LC_50_ was calculated by interpolation of the linear equation. The LC_50_ for the 4 cell lines is listed in Table 2.

LC_50_ values of CP on all the cell types fall between 2.5 and 5 mg/ml and survival rates of the cells treated with 2.5 mg/ml and 5 mg/ml of CP were compared and illustrated in Figure 2. When the cells were treated with 2.5 mg/ml, which is lower than all their LC_50_, the cells demonstrated 40–80% survival rates depending on their susceptibility. When they were treated with 5 mg/ml, which is higher than the LC_50_ of all the cells, MCF-7 and ZR-75-1 cells (both ER(+) PR(+)), demonstrated about 40–50% survival, while MDA-MB-231 and SK-Br-3 (both ER(−) PR(−)), demonstrated only about 10% survival.

### 3.2. Time Course of Cell Survival after Treatment of CP

To test a longer cell survival, the effect of crude extract of CP on the cells was tested for a period of 3 days. The cells were treated with the LC_50_ concentration of the crude extract in a serum-free medium (see Materials and Methods) and were tested with WST based proliferation/cytotoxicity assay (CCK8) at 24, 48, and 72 hours after initial treatment. The viable cell numbers were plotted against the cell number at time zero before drug treatment (Figure 3). The cell number of the treated cells showed time dependent decrease in MDA-MB-231 cells and SK-Br-3 cells (both ER PR negative), while in the case of MCF-7 cells, the decrease in cell number was observed up to 24 hours and then they replenished with increase in cell numbers by 48–72 hours.

### 3.3. Cytotoxicity: Comparison of CP-Treated Conditioned Medium to Direct CP Treatment

The breast cancer cells were known to alter the environment of their distant niche by extracellular secretions containing nucleic acid or protein to promote their survival or metastasis. To test whether treatment by CP to the cells would alter the biomolecular content of the medium to influence cytotoxicity, the CP treated conditioned medium (sup-CP) was collected and supplemented to the naïve cells. These were compared to the effect of direct treatment (dir-CP) on the naïve cells and the effect of 24-hour conditioned medium (sup-Control). We used a similar concentration of CP to be able to compare the cytotoxicity among cells. The MCF-7 cells, because of their higher LC_50_, did not yield significant cytotoxicity when CP was added about 3 mg/ml. For MDA-MB-231 cells and ZR-75-1 cells, the sup-CP treatment does not result in significant cytotoxicity compared to direct CP treatment. For SK-Br-3, direct treatment yielded a much better effect than the CP treated conditioned medium (Figure 4).

### 3.4. The CP Crude Extract as Well as the CP-Treated Conditioned Medium Inhibited the Migration of MDA- MB-231 Cell and MCF-7 Cells in Scratch Assay

The effect of CP treatment on cell migration was tested using the scratch assay. The treatments were similar to the cytotoxicity study. The CP direct treatment (dir-CP), as well as the CP conditioned medium treatment (sup-CP), inhibited the cell migration of two Her2(-) cells lines, MDA-MB-231 and MCF-7, in a 24 hr scratch assay. When treated with 3 mg/ml crude extract, the MDA-MB-231 showed a significant reduction in the cells within the scratched area, demonstrating an inhibition to the cell migration (see Figure 5, dir-CP, and also Figure 6). In the case of MCF-7 cells, the direct CP treatment resulted in reduced cell migration even though it did not render significant cytotoxicity at 3 mg/ml. Therefore, the CP treatment in MCF-7 inhibits migration without enhancing significant cytotoxicity.

The CP extract and sup-CP both inhibited the migration of MCF-7 after 5 days of treatment, while the cells in untreated control or sup-Control had covered up the scratch area with significant migration. For MDA-MB-231, the inhibition was insignificant in the sup-CP treatment after 5 days (Figure 6).

### 3.5. CP-Treated Conditioned Medium (sup-CP) Inhibited the Migration Better than the Direct CP Crude Extract (dir-CP) in MDA-MB-231 cell and SK-Br-3 Cells in Scratch Assay

According to Figures 6(a) and 6(b), in MDA-MB-231 cells, although some of the effects might be caused by the cytotoxicity of the crude extract, as 3 mg/ml was about the concentration of the LC_50_ for these cells, it is interesting that the CP treated conditioned medium (sup-CP) further inhibited the migration than direct treatment (dir-CP). In the case of SK-Br-3 cells, similar to MDA-MB-231 cells, the inhibitory effect of CP-treated conditioned medium on the migration of SK-Br-3 cells is significantly better than the direct CP treatment group (see Figure 6(b), SK-Br-3). A similar dose results in equal or even worse cytotoxicity in the direct CP treatment group. Therefore this discrepancy was not a result of cytotoxicity.

### 3.6. The MMPs and Caspase Expressions: Western Blots

To test the signaling pathways that were responsible for the cytotoxicity and cell migration, the proteins extracted from the naïve cells after treatment with direct CP, or CP-conditioned medium, were tested with Western blot using antibodies against caspase-9, cleaved caspase-3 (proteins involved in the apoptotic pathway), MMP-2, and MMP-9, (proteins responsible for cell migration).

For MDA-MB-231 cells, the expression level of MMP-9 protein was not regulated, while those of MMP-2 were regulated upon CP treatments. Both direct treatment (dir-CP) and CP conditioned medium (sup-CP) yield significantly reduced MMP-2 protein expression when compared to the sup-Control (see Figure 7, MDA-MB-231). However, there was no significant difference between the dir-CP and the sup-CP groups. The cleaved caspase-3, the active form, was also downregulated for the two groups when compared to the control group.

MCF-7 cells showed reduced MMP-2 expression in the sup-CP group. However, as both the dir-CP and sup-CP inhibited the migration (see Figure 5, MCF-7), the antimigration effect may not be solely contributed by the MMP-2 modulation. MMP-9 levels remained unregulated, so was the cleaved caspase-3 protein. Expression of cleaved caspase-3 was reduced when treated with dir-CP in comparison to its respective control (*t*-test, *p* = 0.11) but was not significant. In the case of SK-BR-3 cells, all the protein levels tested were not significantly altered (see Figure 5, SK-BR-3). Levels of cleaved caspase-3 showed a mild increase in the dir-CP treatment when compared to the treatment with CP conditioned medium (*t*-test, *p* = 0.20).

### 3.7. Sensitivity of ER(-) PR(-) Cells to CP Is Not Correlated to Caspase-3

When the CP treated cells were collected and examined, the levels of the caspase-9 and cleaved caspase-3 were not regulated except for the MDA-MB-231 cells (Figure 7). Usually, cells undergoing apoptosis show upregulated expression of activated caspase-3 and in our study, MDA-MB-231 cells show a reduced expression of this protein. Hence, we could infer that CP mediated cellular damage was not caspase-3 dependent and further tests on other signaling pathways such as Sphingosine-1-phosphate [17] or Poly(ADP-ribose) polymerase (PARP) [12] might shed light on the molecules that regulate the cytotoxicity in these ER(-) PR(-) cells (MDA-MB-231 cells).

## 4. Discussion

Breast cancers can be subtyped according to their genotypic variations and metastasis characteristics. The ability of the drug against various types of breast cancer cells differs significantly due to alteration in the metabolic outcome of genotypic characteristics [18, 19]. In this paper, we used 4 different cell lines with varying genotypes to demonstrate a correlation among them with respect to sensitivity towards treatment with CP extract. The following experiments and observations were attempts to correlate the cytotoxicity, migration behavior, and signaling molecules to the genomic composition and responsible signaling molecules. After this, we further made an array of genotypes versus phenotypes to find out their correlation.

Cytotoxicity studies clearly showed that ER(-) PR(-) cells are more sensitive to the CP treatment in terms of direct cytotoxicity. The LC_50_ of these cells are in the increasing order MDA-MB-231<SK-Br-3<ZR-75-1<MCF-7<184A1 (Table 2). If we use the criteria of whether this cell line could be killed significantly to about 20% of untreated cells, the two ER(-) PR(-) cells were sensitive while the double positive cells are resistant to CP treatment (summarized in Table 3). This is the same with other criteria, where cytotoxicity of CP extract after 24–72 hrs was tested (see Figure 3, summarized in Table 2). This demonstrated that the cytotoxicity of CP extract on the breast cancer cells lines is gene type dependent, and ER & PR phenotypes are correlated with the CP cytotoxicity.

Our comparative analysis on inhibition of migration revealed a correlation between Her2(-) phenotypes and partly regulated by MMP-2 level via autocrine fashion. CP crude extract, when administered directly or in a conditioned medium, inhibited the migration of MDA-MB-231 cells and MCF-7 cells (both Her(-)) (summarized in Table 4). SK-Br-3 supplemented with sup-CP did not show significant inhibition of migration compared to its corresponding control (Figure 5). But inhibition was significantly more when compared to dir-CP (Figure 5). Therefore, this inhibition of migration seemed to be correlated to the Her(-) phenotype. Further, the MMP-2 levels were downregulated in MDA-MB-231 cells and MCF-7 cells for the respective treatments. Though migration of Her(-) cells was inhibited by both dir-CP and sup-CP, the corresponding reduction in MMP-2 expression was not observed in dir CP, but MMP-2 expression was reduced in sup-CP treated cells. This clarifies that factors that regulate MMP-2 reduction was present only in CP treated conditioned medium which contains extracellular vesicles/exosomes (Comparison was illustrated in Table 4).

*Cynanchum paniculatum* is a significant part of traditional Chinese medicine where different parts of the plant were utilized to treat a number of ailments. Biological properties rendered by CP were through the bioactive polyphenolic compounds constituted in different plant parts of CP. According to Weon et al., Paeonol is the major constituent present in the 80% methanol extract of roots of *Cynanchum paniculatum* Kitagawa (Asclepiadaceae), [10]. Paeonol is a major phenolic component of Moutan cortex Radicis, a traditional Chinese Medicine. It induces breast cancer cell apoptosis through the regulation of Bcl-2/Bax/caspase 3 signaling, or the CXCR3-B/CXCL4 signals [20, 21]. Paeonol synergizes with Epirubicin on the reduction of breast cancer cells growth via inhibiting PARP, Bax, caspase-3 and also by inhibition of p38/JNK/ERK MAPKs [22]. It was also reported that it could reverse paclitaxel resistance by upregulating the expressions of the adenosine-triphosphate binding cassette transporter proteins [23]. Therefore, it is reasonable to infer that the observed cytotoxicity effect of CP must be mediated through this compound. However, our data did not demonstrate downregulated caspase-3 expression, which suggests that multiple compounds might be responsible within the CP extract, and the compound in our extract responsible for the observed activities may not necessarily be Paeonol.

Until now, more than 440 compounds have been isolated and identified from plants that were classified under genus Cynanchum. These include C21 steroids, steroidal saponins, benzenes and derivatives, alkaloids, flavonoids, terpene, and so on [5]. The chemical composition of CP was previously extracted and single compounds were successfully isolated [2, 3, 11, 24, 25]. Among these compounds cynatratoside B exhibited potent inhibitory activities against HL-60, HT-29, PC-3, and MCF-7 cell lines and cynapanoside D-G displayed moderate cytotoxicity against the four cell lines [11].

Our results demonstrate the ER(-) PR(-) cells are highly sensitive towards CP extract, and comparatively less sensitivity was shown by MCF-7 & ZR-75-1 which expresses estrogen and progesterone receptors (ER & PR). These two receptors and their metabolic role towards cancer development are reported extensively that they play a key role in breast cancer development and their expression was high in the breast cancer cells than the normal human breast cells [26]. ER was well established for its oncogenic property through activation of MAPK pathway resulting in the upregulation of various growth factor receptors that can progress the cancer development. This information about the ER supports the LC_50_ value which is higher for the ER(+) PR(+) cells (MCF-7 & ZR-75-1). The estrogen receptors play a significant role in cancer survival and progression through PI3K/AKT/mTOR pathway [27]. This leads to the focus on the inhibition of these pathways to counter the growth of ER(+) breast cancer cells. Similar to ER, PR also plays a significant role in cancer progression through rapid activation c-Src and ERK2 in breast cancer cells leading to MAPK pathway mediated cancer cell growth. Hence, it was postulated that therapeutic strategies against ER(+) PR(+) breast cancer cells are targeted towards the downregulation of the MAPK/PI3K pathway [28]. The observations clearly depicted the cytotoxicity of CP on ER(-) PR(-) cells (MDA-MB-231) which shows that the molecular target for CP was apparently not similar to the mechanism in ER(+) PR(+) cells. Also, it can be assumed that whichever molecular mechanism the CP targets, the absence of estrogen and progesterone receptors and their downstream signaling is a prerequisite.

Though we found that MCF-7 cells were able to overcome the cytotoxic potential of CP extract, the cell migration property of this cell type was significantly reduced by sup-CP treatment and we could infer that the lack of growth factor receptor gene (Her2(-)) is one of the major reasons, because the ability of MDA-MB-231 cells (also Her2(-)) to migrate was reduced by sup-CP treatment. Her2 upregulation has shown to be linked to higher mortality, recurrence, and high metastatic potential of many cancer cells, including breast cancers. This was achieved by upregulation of an array of pathways which includes PI3K/Akt, Ras/MEK/ERK, and JAK/STAT pathways [29]. Surprisingly, the activity of sup-CP was significantly high compared dir-CP against migration of MDA-MB-231 cells. The fact that altered molecular profile of the conditioned medium, especially the exosome content, due to CP treatment might have shown the primal effect than polyphenols present in the CP extract. Corresponding results were obtained with western blotting analyses where the expression of MMP-2 in Her2(-) cells (MCF-7 & MDA-MB-231) was significantly reduced. The downregulation was observed in both dir-CP & sup-CP treatment in case of MDA-MB-231 cells and only sup-CP treatment had the effect on MCF-7 cells. This shows the role of extracellular vesicles, especially exosomes, on inhibition of cells migration through regulation of MMP-2 mediated mechanism on both the Her2(-) cells. Ramírez-Ricardo et al. has reported that circulating extracellular vesicles have significantly upregulated the cell migration through MMP-2 overexpression [30]. MMPs possess endopeptidase activity and degrade the extracellular matrix and in turn, the basement membrane which during cancer progression is an important process that helps in cell migration, epithelial to mesenchymal transition (EMT), and metastasis [31]. In the case of breast cancer metastasis, MMP-2 & 9 play a pivotal role [32] and our study showed the polyphenolic compounds from CP extract were directly and through modulation of the extracellular profile has influenced the MMP-2 expression in MDA-MB-231 negatively. However, MMP-9 expression in MCF-7 & MDA-MB-231 cells was not downregulated significantly, and we could infer that bioactive compounds in CP extract possess a specific affinity towards MMP-2.

## 5. Conclusion

In summary, ER(-) PR(-) cells are more sensitive to the CP treatment in terms of direct cytotoxicity, which is not regulated by caspase-3. CP treatment inhibits the migration of Her2(-) cells, and this is correlated partly to MMP-2 regulation via autocrine fashion. This provided an interesting starting point for further study of the signaling pathways that regulate the genotype specific reaction to CP drug treatment. Further, given that the treatment of a triple negative breast cancer phenotype is complicated and challenging, the fact that CP targeted multiple phenotypes of this cell is a very interesting discovery. This illustrates that even the single formulation in Chinese medicine could act on multiple targets to hinder the disease progression.

The CP extract consists a group of single compounds and so it was not surprising that breast cancer cells with different genomic compositions would react very differently upon CP treatment. They were differentially sensitive in terms of cytotoxicity or inhibition of migration. Therefore, it is possible to report that cells were responsive to different single compounds (individually or synergistically) within the extract rather than concluding that the same compound has altered different signaling pathways in different cells. Our study from the water extract of CP demonstrated its anticancer potential and also linked the activities to be specific to genotypes, which provide further evidence to explore the composition of CP and their specific targets to achieve precision in quality assurance and practice of Chinese medicine.

## Figures and Tables

**Table 1:** Breast cancer cell lines with different genomic variations used in this study.

Breast cancer cell lines	ER	PR	HER
MDA-MB-231	−	−	−
SK-Br-3	−	−	+
MCF-7	+	+	−
ZR-75-1	+	+	+

**Table 2:** LC_50_ values of the CP extract on different breast cancer cell lines.

Breast cancer cell lines	LC_50_ (mg/ml)
MDA-MB-231	2.68
MCF-7	5.6
SK-Br-3	3.05
ZR-75-1	3.99
184A1 (control)	6.94

**Table 3:** Differential sensitivity to cytotoxicity is correlated to genomic characteristics.

Breast cancer cell lines	ER	PR	HER	5 mg/ml; 24 hr cytotoxicity less than 20%	3 mg/ml; 72 hr cytotoxicity less than 30%
MDA-MB-231	**−**	**−**	−	***Y***	***Y***
SK-Br-3	**−**	**−**	+	***Y***	***Y***
MCF-7	+	+	−	**N**	**N**
ZR-75-1	+	+	+	N	NA

The different genomic characteristics were listed in an array and compared to two traits (shown in the last two columns). The first result column demonstrated that when the cells were treated with 5mg/ml CP extract, MCF−7 and ZR−75−1 (both ER(+)PR(+)) demonstrated about 40-50% survival, hence the N (no) for this phenotype, while MDA-MB-231 and SK−Br−3 (both ER(−)PR(−)), demonstrated about 10% survival, hence the Y (yes). The second result column summarizes the results whether this cell line could be killed significantly to be less than 30% of control group with 3 mg/ml CP after 72 hours of incubation. The two ER(−)PR(−) cells were sensitive (hence the Y result) while the two ER(+)PR(+) cells are not. For details of the results, please see Figure 2 and respective text.

**Table 4:** Differential sensitivity to migration is correlated to genomic characteristics.

Breast cancer cell lines	ER	PR	HER	Direct CP inhibit migration	Direct CP reduced MMP-2 expression	Sup-CP inhibit migration	Sup-CP reduced MMP-2
MDA-MB-231	−	−	−	**Y**	**Y**	**Y**	**Y**
SK-Br-3	−	−	+	N	N	N	N
MCF-7	+	+	−	**Y**	N	**Y**	**Y**
ZR-75-1	+	+	+	N	N	N	N

The different genomic characteristics were listed in an array and compared to two traits (shown in the right four columns). The first result column demonstrated that when the cells were treated with similar CP extract, MDA-MB-231 and MCF−7 (both HER(−)), the migration of cells were inhibited, hence the Y (yes) for this phenotype, while SK-Br−3 and ZR−75−1 (both HER(+)) were not inhibited, hence the N (no). The second result column summarizes the results demonstrated that when the cells were treated with similar CP extract, only MDA−MB−231 cells demonstrated reduced MMP-2 level. There is no association to the genotypes. The third column demonstrated that when the cells were treated with similar CP extract, MDA−MB−231 and MCF-7 (both HER(−)), the migration of cells were inhibited by treatment of CP-treated conditioned medium, while SK−Br−3 and ZR−75−1 (both HER(+)) were not. The fourth column demonstrated that when the cells were treated with CP-treated conditioned medium, MDA−MB−231 and MCF-7 (both HER(−)), the MMP-2 levels were inhibited, while SK−Br−3 and ZR−75−1 (both HER(+)) were not. For details of the results, please see Figures 5–7 and respective text.

## Data Availability

The Excel data used to support the findings of this study are available from the corresponding author upon request.

## Supplementary Materials

Supplementary Materials All details of statistical analysis are included in supplementary file 1.
