# Supplementary material for: Effects of Water Extract of Cynanchum paniculatum (Bge.) Kitag. on Different Breast Cancer Cell Lines
Source: Evid Based Complement Alternat Med. 2021 May 19;2021:6665949. doi: 10.1155/2021/6665949 (PMC8172293; doi:10.1155/2021/6665949)
Supplement: Supplementary Materials — All details of statistical analysis are included in supplementary file 1. [file 6665949.f1.docx]

**Figure 2**

The purpose of this analysis was to find out to which cell lines CP were sensitive.

Test of normality: Shapiro-Wilk test (p-value << 10^‑4^ for a two-way model (cell line + concentration) for survival.

Method for One-way ANOVA due to violation of normality assumption: Kruskal-Wallis test

Post-hoc pairwise comparisons following Krusakl-Wallace test: Dunn’s method (dunn.test package in R)

Significant level: 0.05

Software: R 4.0 ([www.r-project.org](http://www.r-project.org) R Foundation for Statistical Computing, Vienna, Austria)


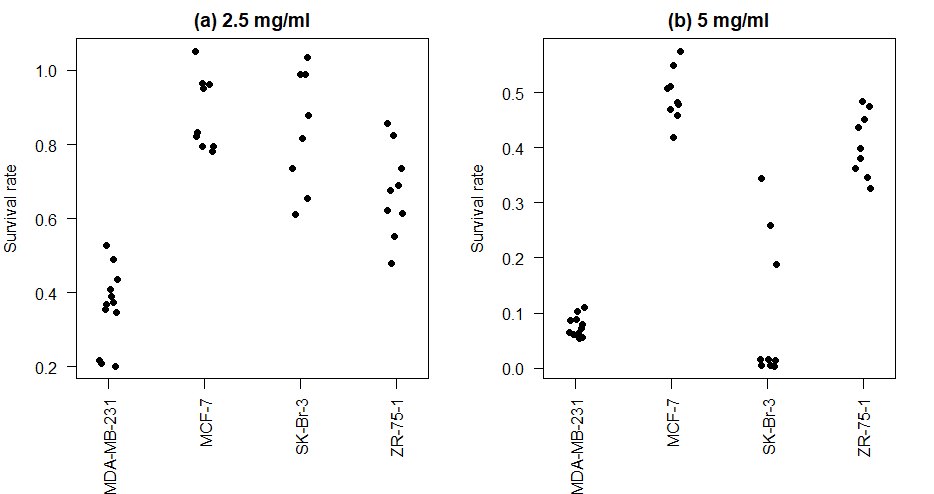


**Figure S1: the survival rates of CP treatment at 2.5 mg/ml or 5 mg/ml.** (a) Survival rate of MDA-MB-231 cell line were significantly lower than that of the MCF-7, SK-Br-3, and ZR-75-1 cell lines with CP at 2.5 mg/ml (p-values = 0.0000, 0.0002, and 0.0319,respectively); (b) The result of pair comparison indicated that there was no survival difference between MCF-7 and ZR-75-1 and between MDA-MB-231 and SK-Br-3 (p-values = 0.3999 and 0.5003, respectively). Rates of Survival of MCF-7 due to CP was evidently different (p-value 0.0001 and 0.0000, respectively) and higher than that of MDA-MB-231 and SK-Br-3 due to CP. Percentage of survival of ZR-75-1 due to CP was also significantly different (p-value was 0.0118 and 0.0035, respectively) and higher than that of MDA-MB-231 and SK-Br-3.

**Table S1: Results of Kruskal-Wallis and Dunn’s pairwise comparison (Z-statistics and p-values) for percentage survival with 2.5 mg/ml CP.** The Kruskal-Wallis test result indicated that the survival rate difference between different cell lines were significant (p-value << 10^-4^). Survival rate of MDA-MB-231 cell line was significantly lower than that of the MCF-7, SK-Br-3, and ZR-75-1 cell lines with CP at 2.5 mg/ml (p-values = 0.0000, 0.0002, and 0.0319, respectively). There was no evident difference in survival rate between any pair of MCF-7, SK-Br-3, and ZR-75-1.

**Table S2 results of Kruskal-Wallis and Dunn’s pairwise comparison for survival rates with 5 mg/ml CP.** The result of pair comparison indicated that there was no survival difference between MCF-7 and ZR-75-1 and between MDA-MB-231 and SK-Br-3(p-values = 0.3999 and 0.5003, respectively). Rates of Survival of MCF-7 due to CP was evidently different (p-value 0.0001 and 0.0000, respectively) and higher than that of MDA-MB-231 and SK-Br-3 due to CP. Percentage of Survival of ZR-75-1 due to CP was also significantly different (p-value was 0.0118 and 0.0035, respectively) and higher than that of MDA-MB-231 and SK-Br-3.

**Figure 3**

The purpose of this analysis was to see whether survival rate change with time for CP treatment at the level of LC_50_. Survival rates were measured at the 0th, 24^th^, 48^th^, and 72^nd^ hour with 2 to 3 replicates.

Since the rates were all 1 at 0 hr, the methods require normality assumption were not appropriate. Non-parametric Kruskal-Wallis test for ANOVA and Dunn’s post-hoc pairwise comparison method were used. P-values were not adjusted for multiple comparison since the adjusted p-values could be too conservative due to such small sample sizes. Correlation between time and survival rate was evaluated using Spearman’s correlation coefficient. All tests used 0.05 as the significant level for p-values. Statistical analyses in this report used R v.4.0 (R Foundation, Vienna, Austria. URL http://www.r-project.org).


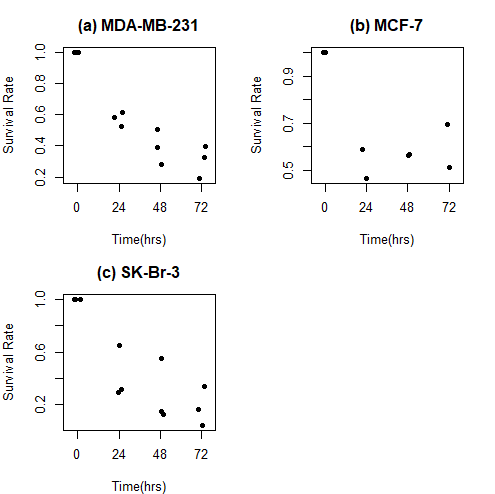


**Figure S2. Time course survival rate after treating with CP at LC^­^_50_ to MDA-MB-231, MCF-7 and SK-Br-3.**

In figure S2(a), it was obvious that survival rate of MDA-MB-231 treated by CP at LC_50_ decreased with time with a negative correlation coefficient -0.9133 (p-value < 10^-4^, see table S3). Survival rates at the 48^th^ and 72^nd^ hour were significantly lower than the initial values (p-value = 0.0166 and 0.0062, respectively. Although test result between survival rates at 0^th^ and 24^th^ hr was not significant (p-value = 0.3048, due to small sample size), but the survival rates between two time points were completely separated.

Figure S2(b) showed that survival rate of MCF-7 treated by CP at LC_50_ dropped at the 24^th^ hour and stayed at nearly the same levels afterwards (or perhaps with a slight upward trend) with completely separated sets of survival rates from the initial values. However, correlation coefficient -0.4909 did not come with a small enough p-value (0.2168). The survival rates did not keep decreasing after the 24^th^ hour. The results of the Dunn’s test did not provide any evidence of difference between survival rates between different time points, although survival rates between 0^th^ hour and 24^th^ hour were nearly at the borderline of significance (0.0646). The test results were shown in table S4.

The survival rate pattern of SK-Br-3 was similar to that of MDA-MB-231, with a negative correlation coefficient -0.7610 (p-value = 0.004) between survival rate and time. This implied that the decrease trend for survival rate with CP at LC_50_ was strongly associated with duration of time after the initial points within 3 days. According to table S5, survival rates at the 48^th^ hour and the 72^nd^ hour were evidently lower than the initial values (p-values were 0.0226 and 0.0116, respectively). Although the survival rates at the 24^th^ hour were not significantly lower than the initial values according to the test result (p-value = 0.1382), the survival rates were all smaller that the initial values.

In conclusion, survival rate due to CP at LC_50_ for MDA-MB-231 and SK-Br-3 gradually and significantly dropped to levels of 0.2-0.3 after 3 days, which for MCF-7, CP at LC_50_ only caused 50% of decrease at the 24^th^ hour, and achieved no more decrease afterwards.

**Table S3 Kruskal-Wallis test, Dunn’s test for survival rate at different time points and the Spearman correlation coefficient between survival rate and time for MDA-MB-231 treated with CP at LC^­^_50_.**

**Table S4 Kruskal-Wallis test, Dunn’s pairwise comparison (Z-statistics and p-values) for survival rates at different time points and the Spearman correlation coefficient between survival rate and time for MCF-7 treated with CP at LC^­^_50_.**

**Table S5 Results of Kruskal-Wallis test, Dunn’s pairwise comparison (Z-statistics and p-values) for survival rate at different time points and the Spearman correlation coefficient between survival rate and time for SK-Br-3 treated with CP at LC^­^_50_.**

**Figure 4**

Test of normality: Shapiro-Wilk test

Method for One-way ANOVA due to violation of normality assumption: Kruskal-Wallis test

Post-hoc pairwise comparisons following Kruskal-Wallace test: Dunn’s method (dunn.test package in R)

Significant level: 0.05

Software: R 4.0 ([www.r-project.org](http://www.r-project.org); R Foundation for Statistical Computing, Vienna, Austria)

**Table S6 Test of normality:** Residuals of three out of four cell lines were not appropriate for the assumption of normality. For consistency in method, non-parametric Kruskal-Wallace method and Dunn’s post-hoc pairwise comparison method were used.


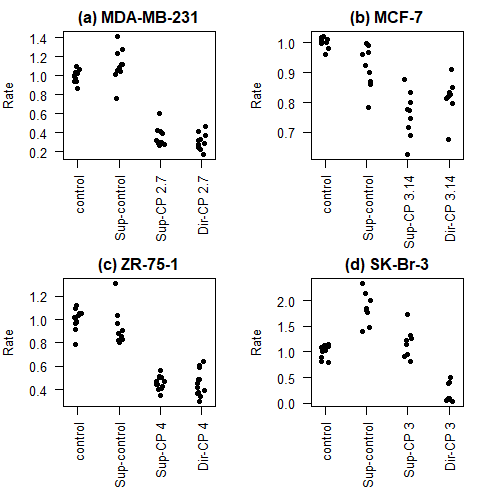


**Figure S3 Cytotoxicity in 4 treatment groups on four different cell lines.** Results in MDA-MB-231, MCF-7, and ZR-75-1, were similar. Both CP treated group had significant cytotoxicity in comparison with both control groups (p-values in table S7) except for MCF-7 the effect difference between sup-control and dir-CP was near the borderline but insignificant (p-value = 0.066). For SK-Br-3, sup-control group had the least cytotoxicity comparing with control, dir-CP, and sup-CP groups (p-value = 0.0025, 0.0000, and 0.0395, respectively). The control group was less cytotoxic than dir-CP group (p-value = 0.0202). sup-CP group appeared to be much more cytotoxic than direct CP group (p-value = 0.0009).

**Table S7 Results of Kruskal-Wallis test and Dunn’s post-hoc pairwise comparisons for cytotoxicity of different treatment groups on MDA-MB-231.** The cytotoxicity rates were significant difference between different groups (p-value < 10^-4^). Cytotoxicity of dir-CP group and sup-CP groups were significantly different from that of the control group (p-values = 0.0003 and 0.0010, respectively). Moreover, cytotoxicity of dir-CP group and sup-CP groups were significantly different from that of the sup-control group (both p-values < 10^-4^, respectively). Cytotoxicity rate difference between two control groups and between two CP-treated group were not evident (p-value = 0.2521 and 0.7525, respectively).

**Table S8 Results of Kruskal-Wallis test and Dunn’s post-hoc pairwise comparisons for cytotoxicity of different treatment groups on MCF-7.** The cytotoxicity rates were significant difference between different groups (p-value << 10^-4^). Cytotoxicity of Dir-CP group and Sup-CP groups were significantly different from that of the control group (p-values = 0.0002 and < 10^-4^, respectively). Moreover, cytotoxicity of Sup-CP groups were significantly different from that of the Sup-control group (p-values = 0.0052). Cytotoxicity rate difference between two control groups was not significant, but closed to borderline (0.0572). The difference between cytotoxicity rate for sup-control and dir-CP-treated group was also closed to the borderline of significance (p-value = 0.0666). The difference between cytotoxicity rate for two CP-treated group was not evident (p-value = 0.3361). ).

**Table S9 Results of Kruskal-Wallis test and Dunn’s post-hoc pairwise comparisons for cytotoxicity of different treatment groups on MCF-7.** The cytotoxicity rates were significant difference between different groups (p-value < 10^-4^). Cytotoxicity of dir-CP group and sup-CP groups were significantly different from that of the control group (both p-values < 10^-4^ ). Moreover, cytotoxicity of dir-CP group and sup-CP groups were significantly different from that of the sup-control group (p-value = 0.0003 and 0.0008, respectively). Cytotoxicity rate difference between two control groups and between two CP-treated group were not evident (p-value = 0.3386 and 0.8078, respectively).

**Table S10 Results of Kruskal-Wallis test and Dunn’s post-hoc pairwise comparisons for cytotoxicity of different treatment groups on SK-Br-3.** sup-control group had the least cytotoxicity comparing with control, dir-CP, and sup-CP groups (p-value = 0.0025, 0.0000, and 0.0395, respectively). The control group was less cytotoxicity than dir-CP group (p-value = 0.0202). sup-CP group appeared to be much more cytotoxic than direct CP group (p-value = 0.0009).

**Figure 5**

Method:

Test of normality: Shapiro-Wilk test

Test of homogeneity: Brown-Forsythe test (onewaytests package in R)

One-way ANOVA for equal variance: ANOVA F-test

One-way ANOVA for unequal variance: Welch F-test (onewaytests package in R)

Post-hoc pairwise comparisons for equal variance: Tukey’s method (userfriendlyscience package in R)

Post-hoc pairwise comparisons for unequal variance: Games-Howell’s method (userfriendlyscience package)

Significant level: 0.05

Software: R 4.0 ([www.r-project.org](http://www.r-project.org); R Foundation for Statistical Computing, Vienna, Austria)


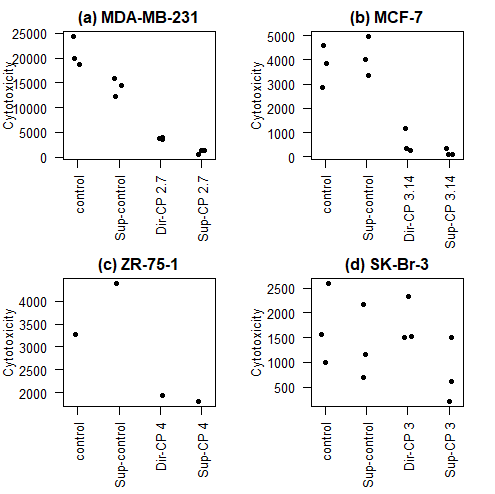


**Figure S4: Cell cytotoxicity**, (a) for MDA-MB-231, it is obvious that cell reductions from both control groups were significant for two CP-treatment groups (p-values in table S-Fig 5.2). Reduction of cells due to sup-CP was greater than that due to direct CP treatment (p-value = 0.016). There were no difference in cell amounts between two control groups (p-values = 0.115); (b) reduction of cells caused by sup-CP and dir-CP were significant (p-value = 0.044 and 0.031, respectively when comparing with direct control, and 0.031 and 0.017, respectively when comparing with sup-control). Difference in reduction of cells caused by two CP-treatment groups was not obvious (p-value = 0.613); (c) According to data of single observation in each treatment group, CP treatments had a trend of reduction in cell amounts; (d) SK-Br-3: no difference in cytotoxicity were visually and statistically detected.

**Table S11 Tests of normality assumption and homogeneity.** No violation of normality assumption was shown. The results of Brown-Forsythe test indicated only SK-Br-3 did not show significant unequal variance between groups. As a result, Welsh’s F-test and Games-Howell’s method were applied to analyses of MDA-MB-231 and MCF-7 data, with standard F-test and Tukey’s post-hoc pairwise comparison method were used for analysis of SK-Br-3 data.

**Table S12 Results of ANOVA and the post-hoc pairwise comparisons between cytotoxicity of different treatment groups on MDA-MB-231 cell lines.** It was significant (p-value = 0.001) that different treatment group had cytotoxicity. Reduction of cells caused by sup-CP and dir-CP were significant (p-value = 0.016 and 0.024, respectively when comparing with direct control, and 0.01 and 0.023, respectively when comparing with sup-control). Reduction of cells caused by Sub-CP was greater than that caused by dir-CP (p-value = 0.016).

**Table S13 Results of ANOVA and the post-hoc pairwise comparisons between cytotoxicity of different treatment groups on MCF-7 cell lines.** It was significant (p-value = 0.0059) that there existed difference in cytotoxicity among different treatment groups. Reduction of cells caused by sup-CP and dir-CP were significant (p-value = 0.044 and 0.031, respectively when comparing with direct control, and 0.031 and 0.017, respectively when comparing with sup-control). Difference in reduction of cells caused by two CP-treatment groups was not obvious (p-value = 0.613)

**Table S14 Results of ANOVA and the post-hoc pairwise comparisons between cytotoxicity of different treatment groups on SK-Br-3 cell lines.** No difference between treatment groups were evident.

**Figure 7**

Test of normality: Shapiro-Wilk test

Test of homogeneity: Brown-Forsythe test (onewaytests package in R)

One-way ANOVA for equal variance: ANOVA F-test

One-way ANOVA for unequal variance: Welch F-test (onewaytests package in R)

Post-hoc pairwise comparisons for equal variance: Tukey’s method (userfriendlyscience package in R)

Post-hoc pairwise comparisons for unequal variance: Games-Howell’s method (userfriendlyscience package)

Significant level: 0.05

Software: R 4.0 ([www.r-project.org](http://www.r-project.org); R Foundation for Statistical Computing, Vienna, Austria)


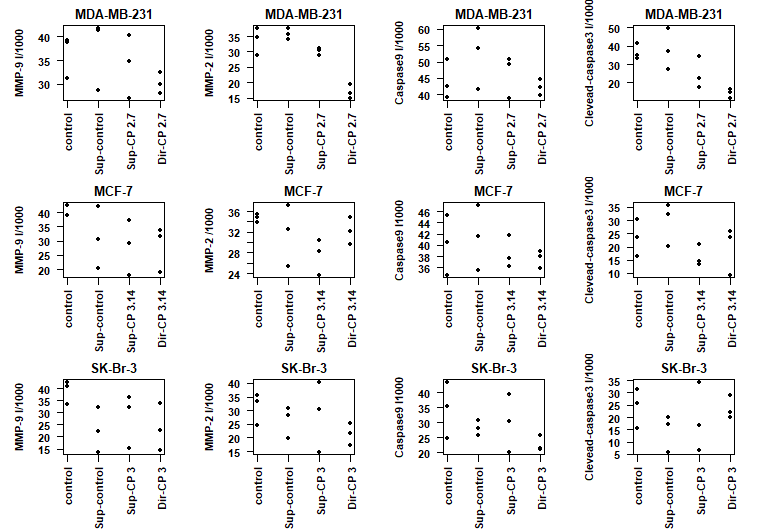


**Figure S5: Protein intensity for each treatment on each cell line.** Only MMP-2 and Clevead-caspase 3 of MDA-MB-231 showed significant difference among different treatment groups according to ANOVA (p-values = 0.0022 and 0.0103, respectively). MMP-2 intensity of sup-CP and dir-CP were both significantly different and lower from that of both controls for MDA-MB-231. MMP-2 intensity of sup-CP were significantly different and lower from that of dir-CP for MDA-MB-231 (sup-control > sup-CP > dir-CP, and Control > dir-CP). Clevead-caspase 3 intensity of dir-CP is significantly different and lower from that of control (Control > dir-CP). For p-values see table S16, S17, S18

**Table S15 p-values for tests of normality, and homogeneity, and the p-values for the one-way ANOVA.** No evidence for violation of normality assumption was detected. Variance of MMP-2 intensity and Clevead-caspase 3 between groups were significantly different (p-values = 0.0022 and 0.0103, respectively). As a result, Welch’s F test and Games-Howell’s post-hoc pairwise comparison method were applied to MMP-2 intensity and Clevead-caspase 3 of MDA-MBA-231. Only MMP-2 and Clevead-caspase 3 of MDA-MB-231 showed significant difference among different treatment groups.

**Table S16 Results for post-hoc pairwise comparisons for intensities of 4 proteins of different treatment groups on MDA-MB-231 cell line.** MMP-2 protein: intensity of dir-CP and sup-CP were both significantly different and lower from that of sup-controls (p-values = 0.002 and 0.037, respectively). Intensity of dir-CP was significantly different and lower from that of control (p-value = 0.03). That is, sup-control > sup-CP > dir-CP, and Control > dir-CP. Clevead-caspase 3: intensity of dir-CP is significantly different and lower from that of control (p-value = 0.012) That is, control > dir-CP.All other comparisons did not show significant difference.

**Table S17 Results for post-hoc pairwise comparisons for intensities of 4 proteins of different treatment groups on MCF-7 cell line.** None of comparisons showed evident difference.

**Table S18 Results for post-hoc pairwise comparisons for intensities of 4 proteins of different treatment groups on SK-Br-4 cell line.** None of comparisons showed evident difference.
